# Supplementary material for: Taming large-scale genomic analyses via sparsified genomics
Source: Nat Commun. 2025 Jan 21;16:876. doi: 10.1038/s41467-024-55762-1 (PMC11751491; doi:10.1038/s41467-024-55762-1)
Supplement: Supplementary file 1 — Supplementary Information [file 41467_2024_55762_MOESM1_ESM.pdf]

Supplementary Materials

Supplementary Note 1.
Our work introduces the concept of sparsifying genomic sequences and processing sparsified sequences in a very fast, efficient, and accurate way. Many attempts were made to reduce the size of the index and alleviate its impact on the overall performance and accuracy. Recent attempts tend to follow one of three key directions: (1) Extracting a smaller number of seeds from genomic sequences, (2) Avoiding the use of some computationally-expensive seeds, and (3) Allowing inexact matches for higher accuracy.

Extracting a number of seeds that is smaller than the number of all possible seeds extracted from a genomic sequence is still challenging. This approach is called sparse seeding[1] or k-mer subsampling[2]. Tools following this direction selectively find a representative *seed* for every group of adjacent seeds. A representative seed can be the seed that 1) has the minimum hash value as in minimizers[3–5], 2) starts with certain DNA alphabets as in Noverlap[1], 3) is located at a predetermined, fixed location as in FEM[6], or 4) its subsequence located at a predetermined location has the minimum hash value compared to these of other subsequences of other seeds as in syncmers[7,8].

In the second direction, one can avoid computationally-expensive seeds that occur frequently in both the reference genome and the read sequences. The high frequency of seeds is due to the repetitive nature of most genomes (about 50% of the human genome is repetitive[9]), which creates a high probability of finding the same seed frequently in a long string of only four DNA letters. The frequency (i.e., the size of the location list) of each seed can be restricted up to a certain threshold to reduce the workload for querying and filtering the seed hits[10–13]. Depending on the coverage (average number of reads covering a base in the reference genome), mapped reads can be overlapping with each other on the reference genome. This means that reads share a large number of similar seeds. To avoid querying genome index multiple times with such query seed, CORA[14] suggests indexing also the read set to improve read mapping execution time. However, CORA increases the memory footprint (~100GB for human genome), and indexing the read set contributes to the total mapping time. Whisper[15] exploits a similar observation and tries to reduce the number of similar seeds extracted from multiple reads by sorting the read set and processing similar reads in groups.

The third direction is to enable finding inexact matches to tolerate genomic variations[16,17] and sequencing errors[18–21] that may affect seed sequences. Spaced seeds[22–28] exclude some bases from each seed following a predetermined pattern. Variable-length seeds (including maximal exact matches (MEMs))[29–33] is another technique that aims to tolerate edit operations by searching for only regions that are located between any two mismatches. Excluded bases help tolerate substitution edits at the excluded locations. Multiple patterns can also be used to exclude different sets of characters belonging to a sequence as in S-conLSH[34]. Multiple very short subsequences can be concatenated together to form a highly sensitive spaced seed without requiring a fixed pattern (e.g., the number of excluded bases may differ in each seed depending on the gap between concatenated subsequences) as in strobemers[35].

There are other techniques, such as S-conLSH[34], conLSH[36], and BLEND[37], that allow generating the same hash value for highly similar seeds or seeds sharing similar context (e.g., neighboring seeds) so that inexact seed matches can still be found using hash tables.

Supplementary Note 2.

We obtain the information below from NCBI RefSeq database: https://ftp.ncbi.nlm.nih.gov/refseq/release/release-notes/archive

Release number	Date of release	Number of distinct organisms	Total number of nucleotide bases	Total number of amino acids sequences	Total number of records
213	July 11, 2022	121'461	3'045'465'416'031	91'290'623'940	321'282'996
205	March 1, 2021	108'257	2'293'291'152'174	76'233'183'903	269'975'565
99	March 2, 2020	99'842	1'865'535'232'080	64'046'042'055	231'402'293
93	March 13, 2019	88'816	1'538'401'021'292	52'033'004'779	192'722'653
87	March 5, 2018	77'225	1'266'924'789'413	40'799'318'419	155'118'991
81	March 6, 2017	68'165	1'022'393'849'190	31'208'765'769	121'954'847
75	March 7, 2016	58'776	807'349'580'822	23'386'816'845	92'936'289
69	January 2, 2015	51'661	594'452'675'642	18'690'872'100	74'127'019
64	March 10, 2014	33'693	407'131'829'420	13'126'329'523	49'538'213

Supplementary Tables

Supplementary Table 1: Index size and indexing time of four state-of-the-art read mappers, mrFAST, minimap2, BWA-MEM, and BWA-MEM2. We index the human reference genome (HG38, GCA_000001405.15), with a FASTA size of 3.2 GB and sort the table by index size. We use the latest version of each read mapper as of 11 November 2022.

Tool	Version	Indexing parameters	Index size	Indexing time
BWA-MEM[38]	Latest (0.7.17-r1188)	default	4.7 GB	49.96 min
minimap2[3]	Latest (2.24-r1122)	-ax map-ont	7.3 GB	3.33 min
mrFAST[11,39]	Latest (2.6.1.0)	default	16.5 GB	20.00 min
BWA-MEM2[40#]	Latest (2.2.1)	default	17 GB	33.36 min

[#]BWA-MEM2's peak memory is exceptionally large (72.3 GB) compared to minimap2 (11.4 GB) when building the index.

Supplementary Table 2. Details of real sequencing read sets used in read mapping evaluation. We calculate the statistics using NanoPlot tool.

**Read set**	**Short reads (Illumina)**	**Accurate, long reads (HiFi)**	**Ultra-long reads (ONT)**
**Accession number and direct download link**	https://ftp-trace.ncbi.nlm.nih.gov/ReferenceSamples/giab/data/AshkenazimTrio/HG002_NA24385_son/NIST_Illumina_2x250bps/reads/D1_S1_L001_R1_001.fastq.gz<br><br>https://ftp-trace.ncbi.nlm.nih.gov/ReferenceSamples/giab/data/AshkenazimTrio/HG002_NA24385_son/NIST_Illumina_2x250bps/reads/D1_S1_L001_R1_002.fastq.gz<br><br>https://ftp-trace.ncbi.nlm.nih.gov/ReferenceSamples/giab/data/AshkenazimTrio/HG002_NA24385_son/NIST_Illumina_2x250bps/reads/D1_S1_L001_R1_003.fastq.gz	https://ftp-trace.ncbi.nlm.nih.gov/ReferenceSamples/giab/data/AshkenazimTrio/HG002_NA24385_son/PacBio_CCS_15kb_20kb_chemistry2/reads/m64011_190830_220126.fastq.gz	https://ftp-trace.ncbi.nlm.nih.gov/ReferenceSamples/giab/data/AshkenazimTrio/HG002_NA24385_son/Ultralong_OxfordNanopore/guppy-V3.4.5/HG002_ONT-UL_GIAB_20200204.fastq.gz<br><br>We consider only the first 2 million reads whose length is greater than or equal 1000 bp (using NanoFilt --length 1000)
**Mean read length**	248.2	18,491.2	15,806.4
**Mean read quality**	8.8	31.3	9.2
**Median read length**	250.0	18,348	5,045

**Median read quality**	31.2	31.8	9.6
**Number of reads**	122,495,089	1,423,276	2,000,000
**Read length N50**	250	18,563	49,093
**STDEV read length**	9.3	2,184.5	34,618.1
**Total bases**	30,405,193,199	26,318,110,120	31,612,763,195
**Longest read length**	250	46,910	1,331,423

Supplementary Table 3: Number of (correctly detected, incorrectly detected, and missed) indels/SNPs and total execution time as provided by Genome-on-Diet using different pattern sequences and Illumina preset (k=21 and w=11).

**Pattern Sequence**	**INDELs / SNPs**			**Execution time (sec)**
	**Correctly detected**	**Incorrectly detected**	**Missed**	
**11**	18'362	**86**	2'862	12'814.20
**110**	19'099	963	2'125	11'129.16
**101**	19'023	1'066	2'201	11'116.24
**011**	19'070	881	2'154	11'034.62
**10**	**19'105**	787	**2'119**	9'178.78
**1001**	18'982	1'370	2'242	9'130.61
**100**	19'025	1'462	2'199	8'086.17
**101001**	18'067	2'531	3'157	**6'351.91**
**100101**	18'194	2'463	3'030	6'565.39
**001101**	18'087	2'736	3'137	6'499.64
**1111100000**	18'840	1'763	2'384	9'926.99
**1001010011**	18'811	1'740	2'413	9'936.54

Supplementary Table 4: Number of (correctly detected, incorrectly detected, and missed) indels/SNPs and SVs along with the total execution time as provided by Genome-on-Diet using different pattern sequences and HiFi preset (k=19 and w=19).

Pattern Sequence	INDELs / SNPs			SVs			Execution time (sec)
	Correctly detected	Incorrectly detected	Missed	Correctly detected	Incorrectly detected	Missed	
**11**	16'731	1'964	4'493	180	11	36	42'494.66
**10**	17'795	2'028	3'429	193	57	23	37'787.72
**100**	14'042	1'822	7'182	141	57	75	15'080.8

Supplementary Table 5. Total execution time (in seconds) and peak memory footprint (in GB) of minimap2 and Genome-on-Diet when performing read mapping.

		Total execution time (sec)		Peak memory footprint (GB)	
	**w**	**minimap2**	**Genome-on-Diet**	**minimap2**	**Genome-on-Diet**
**Illumina k=21**	**5**	68,140.41	12,645.83	23.83	13.75
	**7**	47,715.92	10,566.52	22.98	13.25
	**9**	37,105.37	9,485.03	22.72	13.24
	**11**	30,864.51	8,856.22	14.32	8.96
	**13**	26,603.73	8,385.16	14.33	8.67
	**15**	23,263.23	7,992.77	14.14	8.70
	**17**	21,156.7	7,726.22	13.93	8.67
	**19**	19,529.95	7,587.04	13.91	8.70
	**w**	**minimap2**	**Genome-on-Diet**	**minimap2**	**Genome-on-Diet**
**HiFi k=19**	**7**	72,999.4	41,040.66	47.25	22.54
	**9**	62,956.39	38,945.16	45.88	22.63
	**11**	54,868.53	39,592.18	37.60	18.11
	**13**	49,188.52	38,631.53	36.96	17.70
	**15**	45,246.61	37,292.82	36.84	17.77
	**17**	42,459.01	36,554.15	36.03	17.82
	**19**	40,449.78	35,922.45	35.92	17.91
	**w**	**minimap2**	**Genome-on-Diet**	**minimap2**	**Genome-on-Diet**

ONT k=15	**6**	276,481.5	44,047.18	42.09	49.65
	**8**	197,671.76	48,613.49	37.42	48.52
	**10**	152,426.34	39,410.14	32.72	47.88
	**12**	131,038.03	35,369.44	33.29	48.02
	**14**	112,813.18	29,781.91	31.78	43.47
	**16**	99,198.12	28,176.83	30.51	41.19
	**18**	88,116.78	22,413.19	30.79	40.99

Supplementary Table 6. Number of (correctly detected, incorrectly detected, and missed) indels/SNPs, total execution time, peak memory footprint, and storage footprint provided by Genome-on-Diet, minimap2, and Bowtie2 using Illumina presets. We use two best configurations for Bowtie2: very fast mapping with a small index and very sensitive mapping with a large index.

**Pattern**		**11**	**10**	**minimap2**	**Bowtie2 (very fast, small index)**	**Bowtie2 (very sensitive, large index)**
**INDELs / SNPs**	**Correctly detected**	18'362	19'105	18'178	17'803	17'664
	**Incorrectly detected**	86	787	14	56	46
	**Missed**	2'862	2'119	3'046	3'421	3'560
**Execution time (sec)**		12'814.2	9'178.78	31'358.75	74'613.77	344'820.58
**Peak memory footprint (GB)**		13.8	8.615	14.376	4.14	5.52
**Storage footprint (GB)**		0	0	0	4.2	5.7

Supplementary Table 7: Performance, peak memory footprint, and storage usage of kraken2 for performing containment indexing.

**Indexing Time (sec) (User+Sys)**	**Indexing Memory (GB)**	**Indexing Storage (GB)**
638'986	69.5	192

Supplementary Table 8: Accuracy of KMC3+CMash and Genome-on-Diet in measuring the similarity between two genomic data sets.

		CAMI Low	CAMI High
**Metalign (ground truth)**	**Truly rejected strains/contigs**	13'767'912	13'732'781
	**Truly accepted strains/contigs**	408	35'539
**KMC3+CMash**	**Falsely accepted strains/contigs**	0 (0%)	0 (0%)
	**Truly accepted strains/contigs**	408 (100%)	35'539 (100%)
**Genome-on-Diet (k28, w40)**	**Falsely accepted strains/contigs**	63 (0.000005%)	0 (0%)
	**Truly accepted strains/contigs**	408 (100%)	35'539 (100%)

Supplementary Table 9: Performance, peak memory footprint, and storage usage of BinDash for performing both containment indexing and k-mer intersection.

	Indexing Time (sec) (User+Sys)	Indexing Memory (GB)	Indexing Storage (GB)	k-mer Intersection Time (sec)	k-mer Intersection Memory (MB)	k-mer Intersection Storage (MB)
**CAMI Low**	0	0	0	10'163	22.3	6.6
**CAMI High**	0	0	0	9'604	22.1	6.6

Supplementary Table 10: Containment search accuracy of BinDash.

		CAMI Low	CAMI High
**Metalign (ground truth)**	**Truly rejected strains/contigs**	13'767'912	13'732'781
	**Truly accepted strains/contigs**	408	35'539
**BinDash**	**Falsely accepted strains/contigs**	1'816'336 (13.19%)	1'404'528 (10.23%)
	**Truly accepted strains/contigs**	261 (63.97%)	35'539 (100%)
	**Falsely rejected strains/contigs**	147	0

Supplementary Table 11: L1 norm error, the sum of the absolute differences between the true (by Metalign) and predicted abundances, at the species level for different containment index and taxonomic profiling algorithms.

Containment Indexing Algorithm	Taxonomic Profiling Algorithm	CAMI Low	CAMI High
**KMC3+CMash**	**Metalign**	0	0
	**Genome-on-Diet**	0.0816	0.00272
**Genome-on-Diet**	**Metalign**	0	0
	**Genome-on-Diet**	0.134	0.00272

Supplementary Table 12: Description of the meaning of each mapping quality (MAPQ) value provided by Genome-on-Diet

MAPQ value	Description
**60**	Unique mapping, no secondary alignment.
**6-59**	Based on the quality difference between the best alignment and second-best alignment, where 6 is assigned for the lowest quality difference and 59 is assigned for the highest quality difference.<br><br>`MAPQ = 54 * identity * (dp_max - dp_max2) / (len * match_score - dp_max2) + 5`<br><br>Where<br>`identity= (len - (n_ambi + n_diff))/(len - n_ambi)`<br>`len`: Length of the read sequence<br>`n_ambi`: Number of ambiguous bases<br>`n_diff`: Number of mismatches<br>`dp_max`: Alignment score of the best alignment<br>`dp_max2`: Alignment score of the second-best alignment<br>`match_score`: Score value for matching character
**5**	Mapped with the same quality to 2 locations. Only the primary alignment has a MAPQ of 5 and other alignments of the read are secondaries and have a MAPQ of 0.
**4**	Mapped with the same quality to 3 locations. Only the primary alignment has a MAPQ of 4 and other alignments of the read are secondaries and have a MAPQ of 0.
**3**	Mapped with the same quality to 4 locations. Only the primary alignment has

	a MAPQ of 3 and other alignments of the read are secondaries and have a MAPQ of 0.
**2**	Mapped with the same quality to 5-6 locations. Only the primary alignment has a MAPQ of 2 and other alignments of the read are secondaries and have a MAPQ of 0.
**1**	Mapped with the same quality to 7-9 locations. Only the primary alignment has a MAPQ of 1 and other alignments of the read are secondaries and have a MAPQ of 0.
**0**	Mapped with the same quality to 10 or more locations, or unmapped reads, or secondary alignment.

Supplementary Table 13: Speedup provided by the vectorized implementation of seed extraction over its non-vectorized implementation when processing the complete human genome chromosome 1 and using different k-mer lengths (*k*) and minimizer window sizes (*w*).

	**k=15, w=12**	**k=19, w=19**	**k=21, w=11**	**k=28, w=40**
**Speedup**	1.71x	1.87x	1.66x	2.24x

Supplementary Table 14: Execution time in seconds for sorting a number of locations using three different sorting algorithms for three different data types and different k-mer lengths and window sizes.

	**Number of to-be-sorted locations**	**Radix sort**	**Merge sort**	**Heap sort**
**Illumina (k21, w11)**	1,703	259.07	141.03	165.8
**HIFI (k19, w19)**	14,940	138.22	129.64	143.74
**ONT (k15, w12)**	93,083	282.98	287.14	380.78

Supplementary References

1. Frith, M. C., Noé, L. & Kucherov, G. Minimally-overlapping words for sequence similarity search. *Bioinformatics* (2020) doi:10.1093/bioinformatics/btaa1054.
2. Benoit, G. *et al.* SimkaMin: fast and resource frugal de novo comparative metagenomics. *Bioinformatics* **36**, 1275–1276 (2020).
3. Li, H. Minimap2: pairwise alignment for nucleotide sequences. *Bioinformatics* **34**, 3094–3100 (2018).
4. Roberts, M., Hayes, W., Hunt, B. R., Mount, S. M. & Yorke, J. A. Reducing storage requirements for biological sequence comparison. *Bioinformatics* **20**, 3363–3369 (2004).
5. Schleimer, S., Wilkerson, D. S. & Aiken, A. Winnowing: local algorithms for document fingerprinting. in *Proceedings of the 2003 ACM SIGMOD international conference on Management of data* 76–85 (Association for Computing Machinery, New York, NY, USA, 2003).
6. Zhang, H., Chan, Y., Fan, K., Schmidt, B. & Liu, W. Fast and efficient short read mapping based on a succinct hash index. *BMC Bioinformatics* **19**, 92 (2018).
7. Edgar, R. Syncmers are more sensitive than minimizers for selecting conserved k‑mers in biological sequences. *PeerJ* **9**, e10805 (2021).
8. Pellow, D., Dutta, A. & Shamir, R. Using syncmers improves long-read mapping. *bioRxiv* 2022.01.10.475696 (2022) doi:10.1101/2022.01.10.475696.
9. Lander, E. S. *et al.* Initial sequencing and analysis of the human genome. *Nature* **409**, 860–921 (2001).
10. Jain, C. *et al.* Weighted minimizer sampling improves long read mapping. *Bioinformatics* **36**, i111–i118 (2020).
11. Xin, H. *et al.* Accelerating read mapping with FastHASH. *BMC Genomics* **14 Suppl 1**, S13 (2013).
12. Xin, H. *et al.* Optimal seed solver: optimizing seed selection in read mapping. *Bioinformatics* **32**, 1632–1642 (2016).
13. Jain, C., Rhie, A., Hansen, N. F., Koren, S. & Phillippy, A. M. Long-read mapping to repetitive reference sequences using Winnowmap2. *Nat. Methods* (2022) doi:10.1038/s41592-022-01457-8.
14. Yorukoglu, D., Yu, Y. W., Peng, J. & Berger, B. Compressive mapping for next-generation sequencing. *Nat. Biotechnol.* **34**, 374–376 (2016).
15. Deorowicz, S., Debudaj-Grabysz, A., Gudyś, A. & Grabowski, S. Whisper: read sorting allows robust mapping of DNA sequencing data. *Bioinformatics* **35**, 2043–2050 (2019).
16. Ho, S. S., Urban, A. E. & Mills, R. E. Structural variation in the sequencing era. *Nat. Rev. Genet.* **21**, 171–189 (2020).
17. Poplin, R. *et al.* A universal SNP and small-indel variant caller using deep neural networks. *Nat. Biotechnol.* **36**, 983–987 (2018).
18. Wick, R. R., Judd, L. M. & Holt, K. E. Performance of neural network basecalling tools for Oxford Nanopore sequencing. *Genome Biol.* **20**, 129 (2019).
19. Wang, Y., Zhao, Y., Bollas, A., Wang, Y. & Au, K. F. Nanopore sequencing technology, bioinformatics and applications. *Nat. Biotechnol.* **39**, 1348–1365 (2021).
20. Robasky, K., Lewis, N. E. & Church, G. M. The role of replicates for error mitigation in

next-generation sequencing. *Nat. Rev. Genet.* **15**, 56–62 (2014).
21. Nix, D. A. *et al.* The stochastic nature of errors in next-generation sequencing of circulating cell-free DNA. *PLOS ONE* vol. 15 e0229063 Preprint at https://doi.org/10.1371/journal.pone.0229063 (2020).
22. Ma, B., Tromp, J. & Li, M. PatternHunter: faster and more sensitive homology search. *Bioinformatics* **18**, 440–445 (2002).
23. Chen, Y., Souaiaia, T. & Chen, T. PerM: efficient mapping of short sequencing reads with periodic full sensitive spaced seeds. *Bioinformatics* **25**, 2514–2521 (2009).
24. Břinda, K., Sykulski, M. & Kucherov, G. Spaced seeds improve k-mer-based metagenomic classification. *Bioinformatics* **31**, 3584–3592 (2015).
25. Girotto, S., Comin, M. & Pizzi, C. Efficient computation of spaced seed hashing with block indexing. *BMC Bioinformatics* **19**, 441 (2018).
26. Burkhardt, S. & Kärkkäinen, J. Better Filtering with Gapped q-Grams. in *Combinatorial Pattern Matching* (ed. Landau, G. M.) 73–85 (Springer Berlin Heidelberg, 2001).
27. David, M., Dzamba, M., Lister, D., Ilie, L. & Brudno, M. SHRiMP2: sensitive yet practical SHort Read Mapping. *Bioinformatics* **27**, 1011–1012 (2011).
28. Sović, I. *et al.* Fast and sensitive mapping of nanopore sequencing reads with GraphMap. *Nat. Commun.* **7**, 11307 (2016).
29. Kokot, M., Dlugosz, M. & Deorowicz, S. KMC 3: counting and manipulating k-mer statistics. *Bioinformatics* **33**, 2759–2761 (2017).
30. Liu, S. & Koslicki, D. CMash: fast, multi-resolution estimation of k-mer-based Jaccard and containment indices. *bioRxiv* 2021.12.06.471436 (2022) doi:10.1101/2021.12.06.471436.
31. Wu, T. D. & Watanabe, C. K. GMAP: a genomic mapping and alignment program for mRNA and EST sequences. *Bioinformatics* **21**, 1859–1875 (2005).
32. Liu, Y., Popp, B. & Schmidt, B. CUSHAW3: sensitive and accurate base-space and color-space short-read alignment with hybrid seeding. *PLoS One* **9**, e86869 (2014).
33. Rossi, M., Oliva, M., Langmead, B., Gagie, T. & Boucher, C. MONI: A Pangenomic Index for Finding Maximal Exact Matches. *J. Comput. Biol.* **29**, 169–187 (2022).
34. Chakraborty, A., Morgenstern, B. & Bandyopadhyay, S. S-conLSH: alignment-free gapped mapping of noisy long reads. *BMC Bioinformatics* **22**, 64 (2021).
35. Sahlin, K. Effective sequence similarity detection with strobemers. *Genome Res.* **31**, 2080–2094 (2021).
36. Chakraborty, A. & Bandyopadhyay, S. conLSH: Context based Locality Sensitive Hashing for mapping of noisy SMRT reads. *Comput. Biol. Chem.* **85**, 107206 (2020).
37. Firtina, C. *et al.* BLEND: A Fast, Memory-Efficient, and Accurate Mechanism to Find Fuzzy Seed Matches. *arXiv [q-bio.GN]* (2021).
38. Li, H. Aligning sequence reads, clone sequences and assembly contigs with BWA-MEM. *arXiv [q-bio.GN]* (2013).
39. Alkan, C. *et al.* Personalized copy number and segmental duplication maps using next-generation sequencing. *Nat. Genet.* **41**, 1061–1067 (2009).
40. Vasimuddin, M., Misra, S., Li, H. & Aluru, S. Efficient Architecture-Aware Acceleration of BWA-MEM for Multicore Systems. in *2019 IEEE International Parallel and Distributed Processing Symposium (IPDPS)* 314–324 (2019).
